# Characterization of *Citrus* *nobilis* Peel Methanolic Extract for Antioxidant, Antimicrobial, and Anti-Inflammatory Activity

**DOI:** 10.3390/molecules26144310

**Published:** 2021-07-16

**Authors:** Anjali Malik, Agnieszka Najda, Aarti Bains, Renata Nurzyńska-Wierdak, Prince Chawla

**Affiliations:** 1 Department of Biotechnology, Chandigarh Group of Colleges, Landran, Mohali 140307, Punjab, India; anjalimalik1624@gmail.com; 2 Department of Vegetable Crops and Medicinal Plants, University of Life Science in Lublin, Doświadczalna Street 51A, 20-280 Lublin, Poland; renata.nurzynska@up.lublin.pl; 3 Department of Food Technology and Nutrition, Lovely Professional University, Phagwara, Jalandhar 144411, Punjab, India

**Keywords:** *Citrus nobilis* peel, methanolic extract, antibacterial activity, antifungal activity, anti-inflammatory activity, DPPH

## Abstract

Currently, the potential utilization of fruits and vegetable waste as a source of micronutrients and antioxidants has increased. The present study, therefore, aimed to determine the antimicrobial and anti-inflammatory activities of *Citrus nobilis* peel extract. A modified solvent evaporation technique was employed for peel extract preparation. For effective utilization of the natural product, quantitative analysis of phenolic compounds was carried out using liquid chromatography and mass spectroscopy technique. Phenolic and flavonoids were present in high amounts, while β-carotene and lycopene were present in vestigial amounts. The antimicrobial efficiency of peel extract was evaluated against four bacterial strains including *Staphylococcus aureus* (MTCC 3160), *Klebsiella pneumoniae* (MTCC 3384), *Pseudomonas aeruginosa* (MTCC 2295), and *Salmonella typhimurium* (MTCC 1254), and one fungal strain *Candida albicans* (MTCC 183), and zone of inhibition was comparable to the positive control streptomycin and amphotericin B, respectively. The extract of *Citrus nobilis* peels showed effective anti-inflammatory activity during human red blood cell membrane stabilization (HRBC) and albumin denaturation assay. The extracts also exhibited 2,2-diphenyl-1-picrylhydrazyl (DPPH) scavenging activity ranging from 53.46 to 81.13%. Therefore, the obtained results suggest that *Citrus nobilis* peel could be used as an excellent source of polyphenols and transformed into value-added products.

## 1. Introduction

The citrus plant consists of 17 species that belong to the family *Rutaceae* and these are found almost everywhere in tropical, subtropical, and temperate regions. Orange, kinnow, mandarins, lemons, and citrons grapefruit are important fruits included in this genera [1]. In ancient times, these were grown by the inhabitants of South Asia including Indonesia and China, however, currently, they are grown everywhere across the world. Brazil and China are the two countries with the largest commercial cultivation of citrus fruits along with 140 more countries [2]. The citrus fruit comprises both pulp and peels and among them pulp weighs 44–46% by weight while peel constitutes 10% of the weight. Citrus fruits are believed to be a good source of vitamin C, which plays an important role in the proper functioning of the human body. It activates many enzymes and plays an important role in cell respiration. Vitamin C is also important for preventing and slowing the progression of many diseases such as wounds, fractures, formation of bruises, and bleeding gums [3].

Phenolic compounds like flavonol and flavanones that are considered to be scarcest in most of the plants are present in one-half of the total mass of the fruit of *Citrus nobilis*. Polyphenolic compounds consist of various classes of bioactive compounds including flavonoids, limonoids, coumarins, phenolic acids, terpenoids, tannins, stilbenes, lignans, and carotenoids. In addition to these phenolic compounds, citrus peel consists of the most important flavonoids such as eriocitrin, hesperidin, and naringin [4]. These compounds play an important role in the ecological, physiological as well as in food and pharmaceutical industries [2]. They also consist of heterocycles such as aromatic rings with hydroxyl groups in their basic structure and exist as glycosides. Flavonoids are likely to be key bioactive compounds in citrus peels, particularly in terms of their anticancer activity as well as in the prevention of infectious and degenerative diseases [2,3]. Furthermore, there is a growing emergence of drug resistance against pathogenic microorganisms which is supposed to be a serious threat to mankind, and therefore it is important to choose the most important antibiotics and to use them properly [5]. There is a constant formation of free radicals in the human body during energy production in the mitochondrial respiratory chain, fertilization, phagocytosis, metabolism. These formed free radicals provoke uncontrolled chain reactions that include lipid peroxidation which leads to the development of cancer, neurological disorders, cardiovascular disease, rheumatoid arthritis, and diabetes [6]. Reactive oxygen species activate the nuclear factors and induce the synthesis of cytokines that are responsible for the development of inflammatory response syndrome. Inflammatory mediators and adhesion molecules are also formed later. Free radicals result in loss of cell functions which later leads to cell death at the site of inflammation [5]. The immune system of all organisms is not sufficient to protect the body against free radicals. Synthetic antioxidants including butylated hydroxytoluene and hydroxyanisole available and consumed have side effects and are thought to be responsible for carcinogenesis and liver damage [7]. To protect living beings from oxidative damage, naturally occurring antioxidants such as Vitamin A, C, and E, carotenoids, flavonoids, and other simple phenolic compounds are preferred [8]. In different studies, various extraction methods are used to extract the bioactive compounds responsible for biological activities, however, they are not much more effective as they may lead to autooxidation of the biological compounds [5,9]. To overcome this problem, the modified solvent technique could be a good approach, as in this process, polyphenolic compounds become dispersed in an organic solvent and accumulate high kinetic energy when they exchange with neighbor molecules to escape from the bonds with another molecule, resulting in separation of molecules from the mass of liquid in the form of vapors along with air [8,9]. Therefore, the present study was conducted to investigate the antimicrobial, antioxidant, and anti-inflammatory activity of methanol extract of *Citrus nobilis* peel.

## 2. Results and Discussion

### 2.1. Characterization of Modified Solvent Evaporated Citrus Nobilis Peel Dry Extract

**Figure 1:**
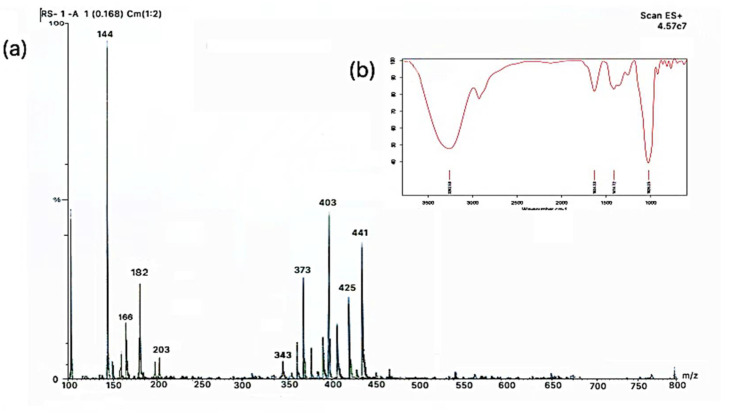
Characterization of *Citrus nobilis* peel extract using (**a**) FTIR spectra; (**b**) LCMS chromatogram.

The functional groups and identification of the bioactive compounds responsible for different biological activities were evaluated using Fourier-transform infrared spectroscopy (FTIR) and liquid chromatography and mass spectroscopy (LCMS) technique and results are represented in Figure 1a,b, and Table 1. Herein, the stretching peak at 3262.58 cm^−1^, 1028.25 cm^−1^, 1414.72 cm^−1,^ and 1634.33 cm^−1^ corresponds to N-H/C-H/O-H stretching of amines and amides, the vibration of C-O in alcohol hydroxyl group, C-O/C-H bending, and diketones [10]. Analyses by LCMS were performed to unequivocally confirm the characteristics identity of polyphenols extracted from peels [11]. Analytical protocol development consisted of running the standard solutions on the two C18-based columns. Using a simple isocratic mobile phase, separation was obtained for the polyphenolic component of peel extract. Although the other peaks eluted promptly, limonene was strongly retained under these conditions and eluted in later runs as carryover. During analysis, gallic acid, myricetin, and resveratrol were not identified at the method detection limits, whereas *p*-coumaric and *trans*-ferulic acids were detected in peel extracts. This could be due to low sensitivity allied to the negative ionization mode. In addition, due to the high selectivity of the method detection, limonene, 4-butoxy phenol, anthocyanin, eupatorine, sinensetin, tangeretin, and nobiletin were detected. Therefore, from the obtained results, *Citrus nobilis* peel could be considered an infrequent source of rutin and/or quercetin, however, remarkably, it could be a rich source of limonene, eupatorine, and nobiletin, which were extracted at quite elevated amounts.

### 2.2. Antimicrobial Activity

**Figure 2:**
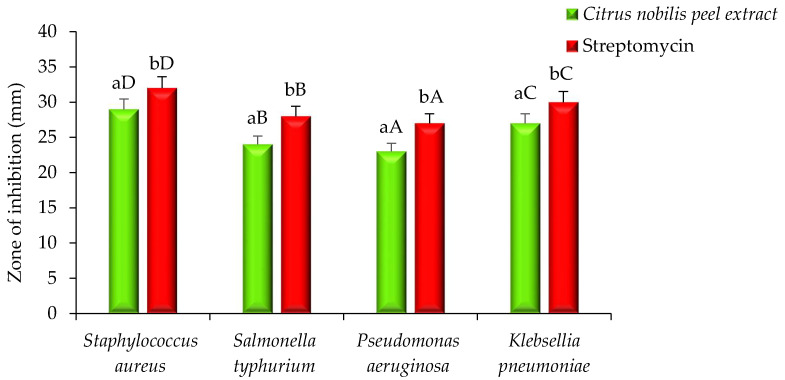
Antibacterial activity (zone of inhibition) of *Citrus nobilis* peel dry extract against Gram-positive and Gram-negative bacteria. Data are presented as means ± SEM (*n* = 3); a,b: Means within the column with different lowercase superscripts are significantly different (*p* < 0.05); A–D: Means within the row with different uppercase superscript are significantly different (*p* < 0.05) from each other.

**Figure 3:**
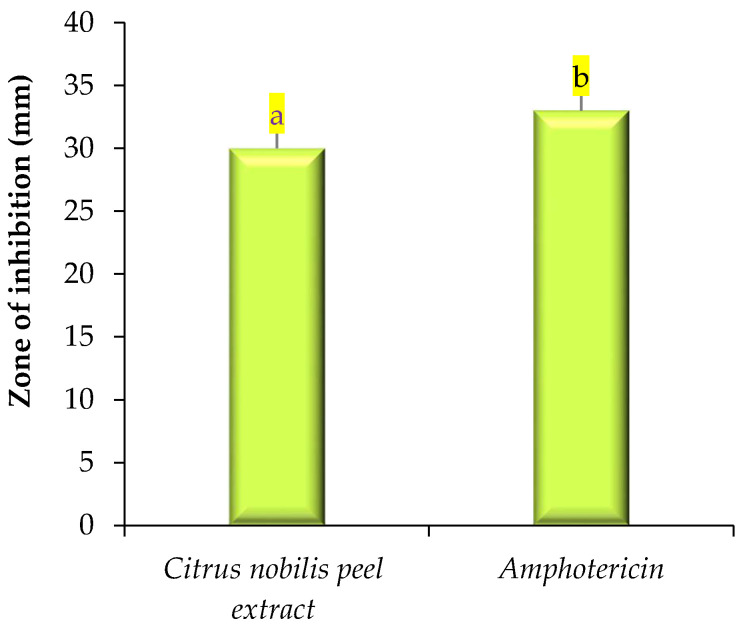
Antifungal activity (zone of inhibition) of *Citrus nobilis* peel dry extract against *Candida albicans.* Data are presented as means ± SEM (*n* = 3); a,b: Means within the column with different lowercase superscripts are significantly different (*p* < 0.05).

The antimicrobial activity of modified solvent evaporated dry extract of *Citrus nobilis* peel was evaluated against both pathogenic bacteria and fungi and results are represented in Figure 2 and Figure 3. The extract showed a significantly (*p* < 0.05) higher zone of inhibition against *Staphylococcus aureus* in comparison with the zone of inhibition against *Klebsiella pneumonae*, *Salmonella typhimurium*, and *Pseudomonas aeruginosa*, respectively. Dry extract of *Citrus nobilis* peel showed the zone of inhibition in the range 23–29 mm, whereas positive control showed a significantly higher zone of inhibition in the range 27–32 mm, respectively. The susceptibility of the extract against Gram-positive bacteria *S. aureus* is due to the presence of a cell wall constituting the formulation of a thick hydrophobic structure that provides a large number of proteins lipids, peptidoglycan, and other biological components present in the extract to pass through the cell membrane. Gram-negative bacteria on the other hand consist of a lipopolysaccharide layer on its outer membrane that results in a change in the hydrophobic properties or mutation in porins and other factors and therefore results in resistance against the extract [8,12,13]. The extract showed good antifungal activity against *Candida albicans* with a zone of inhibition of 30 mm, whereas amphotericin showed a significantly (*p* < 0.05) higher zone of inhibition of 33 mm. The antifungal activity is believed to exist due to the presence of phytochemicals called limonene that diffuse into the cell membrane and make it permeable. The permeability of the phytochemical compounds, therefore, results in leakage into the cell [13]. Our results were in accordance with the findings of Singh et al. [14] Safdar et al. [15] and Saleem et al. [16] who observed the antimicrobial effect of essential oil of *Citrus maxima* Burm. and *Citrus sinensis* (L.), ethanol and methanol extract of *Citrus reticulate* and waste fruit peels extract (orange, yellow lemon, and banana) extracted from different solvents against pathogenic Gram-positive and Gram-negative bacteria and fungi respectively. In this research, the solvent was evaporated at refrigerated temperature, and at this temperature, polyphenolic components dispersed in methanol and molecules of methanol or organic solvent gather enough kinetic energy from its exchange with neighbor molecules to escape from the bonds with another molecule, hence molecules leave the mass of liquids and join the air as a vapor. Our results are well supported by our previous study where we used this process to evaluate the antimicrobial properties of *Pleurotus floridanus*. From this information, we can conclude no methanolic residues were present in peel extract [8].

### 2.3. Time–Kill Kinetics

**Figure 4:**
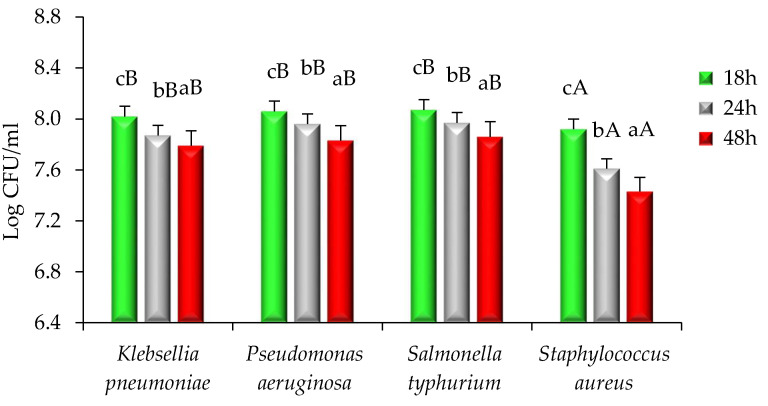
Time–kill kinetics of *Citrus nobilis* peel dry extract against the growth of food pathogenic bacteria during different time intervals. Data are presented as means ± SEM (*n* = 3); a–c: Means within the column with different lowercase superscripts are significantly different (*p* < 0.05); A,B: Means within the row with different uppercase superscripts are significantly different (*p* < 0.05) from each other.

**Figure 5:**
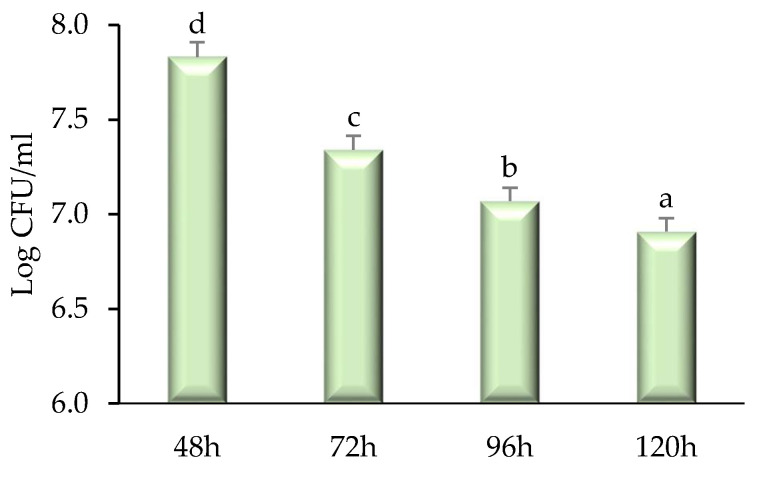
Time-kill kinetics of *Citrus nobilis* peel dry extract against the growth of pathogenic *Candida albicans* during different time intervals. Data are presented as means ± SEM (*n* = 3); a–d: Means within the column with different lowercase superscripts are significantly different (*p* < 0.05).

A time–kill study of modified solvent evaporated dry extract of *Citrus nobilis* peel was performed and results are represented in Figure 4 and Figure 5. With the increase in the time interval, the extract showed a significantly (*p* < 0.05) higher killing effect against both bacteria and fungi taken for study. Herein, among all bacterial strains, *Staphylococcus aureus* showed significantly (*p* < 0.05) lower log CFU/mL value reduction (7.92–7.43) followed by *Klebsiella pneumonia* (8.02–7.79), *Pseudomonas aeruginosa* (8.06–7.83), and *Salmonella typhimurium* (8.07–7.84). The higher reduction in the CFU/mL value of *Staphylococcus aureus* is due to the presence of peptidoglycan that allows antibiotics and peptidoglycan to enter inside the cell resulting in destruction and denaturation of the cell membrane. The Log CFU/mL value for *Pseudomonas aeruginosa* was higher than other microorganisms because the microorganism possesses a resistance mechanism that includes outer membrane permeability restriction, efflux pump, and enzymes that result in the deactivation of antibiotic and bioactive compounds responsible for antimicrobial activities [4,15]. There was a significant (*p* < 0.05) reduction in the Log CFU/mL value of *Candida albicans* (7.83–6.91). The reduction in the value is due to the presence of phytochemicals in the extract that diffuse into the cell membrane of the fungi resulting in cell destruction [17,18].

### 2.4. Estimation of Bioactive Compounds and Their Antioxidant Activity

**Figure 6:**
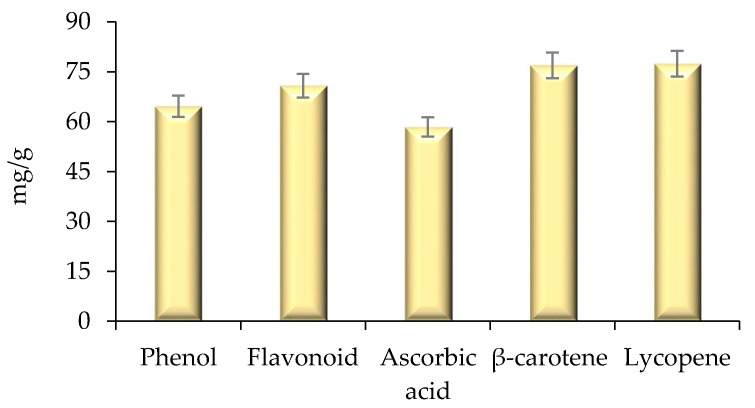
Total phenol, flavonoid, ascorbic acid content, total β-carotene, and lycopene content of modified solvent evaporated *Citrus nobilis* peel extract. Data are presented as means ± SEM (*n* = 3).

**Figure 7:**
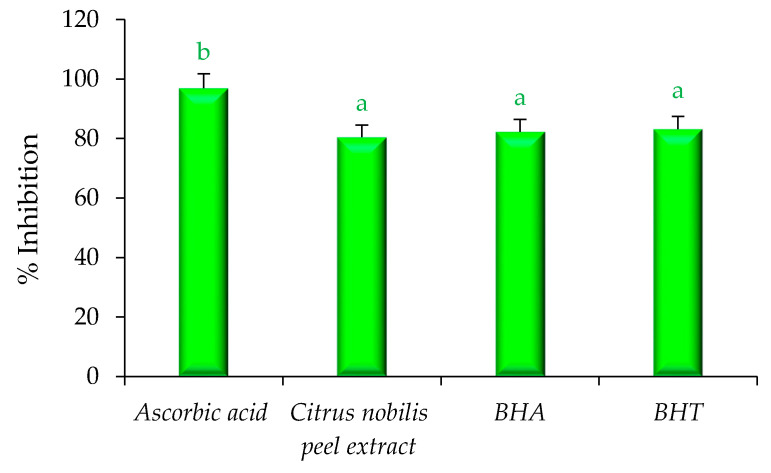
Antioxidant activity of *Citrus nobilis* peel dry extract in comparison with natural and synthetic commercially available antioxidant components. Data are presented as means ± SEM (*n* = 3); a,b: Means within the column with different lowercase superscripts are significantly different (*p* < 0.05).

Herein, the quantification of bioactive compounds phenol, flavonoid, ascorbic acid, lycopene, and carotene was evaluated using a UV-Visible spectrophotometer, and results are represented in Figure 6. The extract showed higher amount of β-carotene (76.92 mg/g), lycopene (77.92 mg/g), flavonoid content (70.82 mg/g), phenol (64.63 mg/g), and ascorbic acid (58.42 mg/g), respectively. The antioxidant activity of modified solvent evaporated extract of *Citrus nobilis* peel was evaluated and the results are represented in Figure 7. Herein, all concentrations of L-ascorbic acid, BHA, BHT, and extract showed a significant (*p* < 0.05) difference in percentage inhibition of DPPH. The extracts exhibited DPPH scavenging activity ranging from 53.46 to 81.13%. Ascorbic acid showed significantly (*p* < 0.05) higher percentage inhibition 96.92%, followed by BHA (82.34%), BHT (83.23%), and extract (81.46%). However, BHT, BHA, and peel extract showed non-significant (*p* > 0.05) differences with each other in terms of percentage inhibition. Therefore, it can be concluded that the ability of peel extract to scavenge free radicals by DPPH scavenging assay was similar to the artificial antioxidants BHA and BHT. In this context, Safdar et al. [14] observed an excellent DPPH free radical scavenging activity of methanolic extract of orange peel. In another study by Do et al. [19] the ethanolic extract of *Limnophila aromatica* peel, and pulp of *Citrus mandarin*, exhibited maximum DPPH free radical scavenging activity. Likewise, Karsheva et al. [20] also observed that ethanol extract of kinnow mandarin peel showed excellent free radical scavenging activity. The explanation may rely on the kinetic release of the antioxidants from the extract. Various intrinsic bioactive components are known to have first-order release kinetics from the extract of *C. nobilis* peel [5].

### 2.5. Anti-Inflammatory Activity

**Figure 8:**
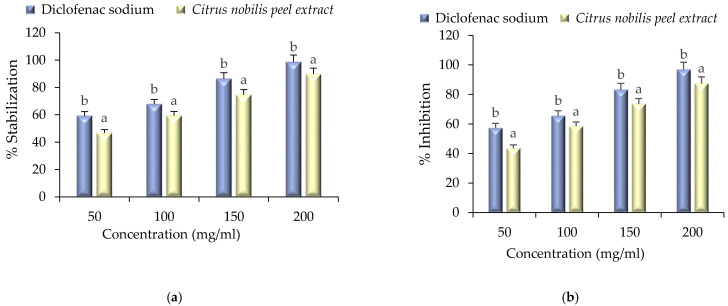
Anti-inflammatory activity of *Citrus nobilis* peel dry extract during (**a**) HRBC membrane stabilization assay and (**b**) albumin denaturation assay. Data are presented as means ± SEM (*n* = 3); a,b: Means within the column with different lowercase superscripts are significantly different (*p* < 0.05).

Anti-inflammatory assay of the extract was performed by HRBC membrane stabilization and albumin denaturation assay and results are represented in Figure 8a,b. Herein, anti-inflammatory activity was compared with standard diclofenac sodium. During membrane stabilization assay and albumin denaturation assay a significant (*p* < 0.05) difference was observed between standard and extract. The percentage stabilization of HRBC membrane stabilization ranged from 46.76 to 89.67% and albumin denaturation ranged from 43.56 to 87.57% respectively. However, standard diclofenac sodium showed a significantly (*p* < 0.05) higher range of stabilization (59.35–98.71%) during HRBC membrane stabilization assay, whereas, during albumin denaturation assay, the % inhibition ranged from 57.42 to 96.96%. The anti-inflammatory activity of the extract was due to the presence of nobiletin, sinenstin, and other bioactive compounds [1]. Furthermore, inflammation and oxidation are closely related, and free radicals that damage the cells lead to inflammation. It is a preventive effort by an organism for removing injurious stimuli alongside inflammation to start a signal for the healing process. The evidence proves that membrane stabilization is an additional mechanism of the anti-inflammatory effect of the extract. The release of lysosomal contents from neutrophils might be inhibited at the inflammation sites for membrane stabilization due to the bioactive compounds present in the extract. The present study is in accordance with the findings of Shin et al. [21], Xiong et al. [22], He et al. [23], and Wang et al. [24] who employed these compounds during inflammation treatment.

## 3. Materials and Methods

Fresh *Citrus nobilis* (Daisy variety) were harvested from a local farm of Mohali, Punjab, India. Analytical reagent grade media including Muller Hinton Agar (MHA), Sabouraud Dextrose Agar (SDA), Nutrient agar, phosphoric acid, methanol was procured from Hi-Media, Private Limited, India. Both Gram-positive and Gram-negative bacterial strains *Staphylococcus aureus* (MTCC 3160) *Klebsiella pneumoniae* (MTCC 3384), *Pseudomonas aeruginosa* (MTCC 2295), and *Salmonella typhmurium* (MTCC 1254) one fungal strain *Candida albicans* (MTCC 183) were obtained from Microbial Type Culture Collection, Chandigarh, India.

### 3.1. Phytochemical Assays

#### 3.1.1. Chemical Reagents

Ethanol, acetonitrile, and methanol of gradient HPLC quality were purchased from Fisher scientific private limited, Mumbai, India. Dimethyl sulfoxide (DMSO, ≥99.9%), 2 N Folin–Ciocalteu’s reagent, 2,2-diphenyl-1-picrylhydrazyl (DPPH), trifluoroacetic acid (TFA, 99%), were procured from Sigma-Aldrich (St. Louis, MO, USA). Ammonium molybdate tetrahydrate, aluminum chloride 6-hydrate, sulphuric acid (95–98%), sodium carbonate anhydrous, tri-sodium phosphate 12-hydrate, butylated hydroxyanisole (BHA), butylated hydroxytoluene (BHT), sodium hydroxide pellets, and sodium nitrite were purchased from Hi-Media Private Limited, Mumbai, India. For LC-MS analysis, methanol, water, and formic acid (LC/MS grade) were procured from Fisher scientific private limited, Mumbai, India. All chemicals and solvents were of “analytical grade”. Phenolic standards such as gallic acid (≥98.0%); *p*-coumaric acid, (≥98.0%); trans-ferulic acid, 98%); rutin; myricetin; resveratrol (≥99%); hesperidin, naringin (≥95%), quercetin, (≥95%) were purchased from Sigma-Aldrich (St. Louis, MO, USA).

#### 3.1.2. Preparation of Peel Extract

*Citrus nobilis* peels were kept for drying in a hot air oven at 30 °C to remove the water content. The dried peels were ground using a mechanical grinder (Sujata, mixer grinder, 900 watts) and the powder thus obtained was used for the preparation of the extract. Herein, the modified solvent evaporation technique was employed for extract preparation. Briefly, 40 g powdered peel was dispersed in 400 mL (1:10 *w*/*v* ratio) of absolute methanol in a conical flask and kept in an orbital shaker (Thermofisher Scientific Pvt. Ltd., Mumbai, India) for 72 h. To obtain the modified evaporated extract, the sample was filtered using Whatman filter paper No. 1 and evaporation of methanol was done at refrigerated temperature (4–7 °C) for 72 h. At refrigerated temperature, polyphenolic components were dispersed in methanol and molecules of methanol collected enough kinetic energy from its exchange with neighbor molecules to escape from the bonds with another molecule, hence molecules leave the mass of liquids and join the air as a vapor. The obtained dry extract was stored at −20 °C in airtight glass tubes for further use [8].

#### 3.1.3. Characterization of Extract

The dry extract of *Citrus nobilis* peel was characterized by Fourier-transform infrared spectroscopy (FTIR) and Liquid chromatography-mass spectrometry techniques. Functional groups present in *Citrus nobilis* peel extract were evaluated FTIR (Bruker Scientific Private Limited, Mumbai, India). Data were obtained in terms of transmittance (55 scans) using air as background in mid-infrared spectral region 4000–600 cm^−1^. The liquid chromatography-mass spectroscopy technique was used for the identification of polyphenolic compounds. Herein, the sample was first filtered through a syringe-driven filter. The system consisted of a diode array detector, Cosmosil 5-C18-MS-II, and analytic column C18-ODS (Waters India Pvt. Ltd. Chandigarh, India). The mobile phase consisted of acetonitrile, trifluoroacetic acid dissolved in double-distilled water. The sample injection volume was 20 µL, the flow rate was 0.8 mL/min, and the temperature of the column was kept at 40 °C. The different polyphenolic compounds were identified by comparing the *m*/*z* value [10].

#### 3.1.4. In Vitro Antimicrobial Activity

In vitro antimicrobial activity against both pathogenic bacteria and fungi including *Staphylococcus aureus*, *Klebsiella pneumonia*, *Pseudomonas aeruginosa*, *Salmonella typhimurium*, and one fungal strain *Candida albicans* was determined by the agar well diffusion method proposed by Bains and Chawla, [5]. Herein, the bacterial strains (1.5 × 10^8^ cells/mL) were inoculated in Muller Hinton agar plate enriched with 4% NaCl and the fungal strain was inoculated in Sabouraud Dextrose agar plate. The plates were allowed to dry and using a cork borer wells were made. The peel extract (10 mg) dissolved in 10 mL of DMSO (8%) was poured (50 µL) into an agar well with the help of a micropipette. To compare the antimicrobial activity of peel extract with a commercially available antibiotic component, streptomycin (1 µg/mL) was used as a standard (positive control) antibacterial agent, whereas, DMSO was used as a negative control. Whereas, for antifungal activity, amphotericin (1 µg/mL) was used as a positive control. The plates containing bacterial strains were then incubated at 37 °C for 24 h while plates containing fungal strain were incubated at 27 °C for 72 h and the zone of inhibition was measured in mm.

*Time-kill* study was performed by the method followed by Majeed et al. [25]. Briefly, 100 µL of peel extract solution was taken for all pathogenic microbial samples after a time interval of 0, 18, 24, and 48 h for the bacterial strain and 48–120 h, respectively for fungal strain. The samples of bacterial strain and fungal strain were spread on plates containing MHA and SDA respectively. The plates were then incubated at 37 °C for 24 h for bacterial strains and 27 °C for 72 h for fungal strains. Log CFU/mL was then calculated for each sample.

#### 3.1.5. Quantification of Phenolics

*Total phenolic content* (TPC). The evaluation of total phenolic content was done by the method described by Najda et al. [26]. Herein, 10 mg/10 mL stock solution of extract was prepared in methanol, and 200 µL of the extract was properly dissolved in 1 mL (1 N) Folin-Ciocalteu reagent and 7.5% (2 mL) sodium carbonate solution. It was then kept undisturbed and constant under dark conditions for 30 min. The absorbance was measured at 760 nm after 30 min of incubation under dark conditions using UV-Visible spectrophotometer (Pharma Test Apparatebau AG, Hainburg, Germany). Gallic acid (GAE) was used as standard and its different concentration was used to plot calibration curve. Total phenolic content was calculated GAE/g.

*Flavonoid content estimation* (FC). The total flavonoid content was evaluated by the method proposed by Bains and Chawla [5]. Dry extract (2 mL) was mixed with 200 µL sodium nitrite (5%) and kept constant for 5 min. Thereafter, 200 µL (10%) aluminum chloride was added and the mixture was mixed evenly using a vortex shaker. The reaction mixture was then kept undisturbed for 6 min and 2 mL (1 M) NaOH was added to it. The absorbance of the reaction mixture was immediately observed at 510. nm using a UV-Visible spectrophotometer. Total flavonoid was calculated by using quercetin (QR) w for the calibration curve.

*Estimation of ascorbic acid* (AA). The ascorbic acid content of *Citrus nobilis* peel dry extract was calculated according to the method proposed by Najda et al. [27]. The extract (100 mg) was mixed with 1% metaphosphoric acid and kept constant for 45 min at 30 °C. The mixture was then filtered through Whatman no.1 filter paper and the filtrate thus obtained was dissolved in 2,6 dichlorophenol. Within 30 min the absorbance of the mixture was measured at 515 nm. The calculation of the total amount of ascorbic acid was done by using L-ascorbic acid as a standard curve.

*Estimation of total β-carotene* (TC) *and lycopene content* (TL). The peel extract (100 mg) was mixed with 10 mL of the acetone-hexane mixture (4:6) and kept undisturbed for 1 min at room temperature. The mixture was filtered using Whatman No. 4 filter paper and absorbance was measured at 453, 505, and 663 nm respectively [28]. The following equation was applied to calculate the total β-carotene and lycopene content.

β-carotene (mg/100 mg) = 0.216A_663_ − 0.304A_505_ + 0.452A_453_ (1)

Lycopene (mg/100 mg) = −0.0458A_663_ + 0.372A_505_ − 0.0806A_453_ (2)

#### 3.1.6. In Vitro Antioxidant Activity

*DPPH free radical scavenging activity.* The total antioxidant activity was evaluated by method followed by Bains and Chawla [5]. The extract of *Citrus nobilis* peel (200 µL) was mixed with 2 mL of 0.1 mM DPPH solution in a test tube and kept for 30 min in dark conditions. After 30 min there was an observed change in color of DPPH from purple to pale yellow. The absorbance was then measured at 517 nm using a UV-Visible spectrophotometer and the percentage of free radical scavenging activity was calculated as follow:

(3) $$\mathrm{Inhibition}\;\left(\%\right)=\left(1-\frac{\mathrm{Ap}}{\mathrm{Aq}}\right)\times 100$$

where: Ap-is the absorbance of the solution; Aq-absorbance of the control (DPPH).

The extract was compared with commercially used butylated hydroxyanisole (BHA) and butylated hydroxytoluene (BHT). The concentration of BHA and BHT were kept equivalent to the concentration of peel extract.

#### 3.1.7. In Vitro Anti-Inflammatory Properties

*HRBC membrane stabilization assay.* To perform HRBC membrane stabilization assay, blood (5 mL) from a healthy volunteer who did not intake nonsteroidal anti-inflammatory drugs (NSAID) for 15 days. The blood was mixed in an equal volume of Alsever solution (20.5 g dextrose, 8 g sodium citrate, 0.55 g citric acid, and 4.2 g sodium chloride in 1000 mL water). The mixture was centrifuged at 3000× *g* for 15 min. The packed cell obtained after centrifugation were washed with isosaline solution. The assay mixture containing 500 µL HRBC suspension, 0.15 M phosphate buffer (1 ml) with pH 7.4, 0.36% hyposaline solution (2 mL), and 500 µL peel extract was prepared. The assay mixture was then incubated at 37 °C for 30 min in Biological oxygen demand (BOD) incubator and after 30 min of incubation, the suspension was centrifuged at 3000× *g* for 20 min. Diclofenac sodium and deionized water were used as positive and negative control. The hemoglobin content present in the supernatant was evaluated by measuring the absorbance at 560 nm using a UV-Visible spectrophotometer [4].

The percentage HRBC membrane stabilization was calculated as follows:

(4) $$\mathrm{Inhibition}\;\left(\%\right)=100-\left(\frac{\mathrm{OP}}{\mathrm{OD}}\right)\times 100$$

where: OP—optical density of the sample; OD—absorbance of control.

*Albumin denaturation assay.* Albumin denaturation assay was performed by preparing a volume of 5 mL of the reaction mixture containing 200 µL of fresh egg albumin, phosphate buffer solution (2.8 mL) with pH 6.4, and 2 mL of peel extract. The reaction mixture was incubated in a BOD incubator at 37 °C for 15 min followed by heating up to 70 °C for 5 min and absorbance was measured at 660 nm. Diclofenac sodium and deionized water were used as positive and negative control. Percentage inhibition by albumin denaturation assay was calculated as follow:

(5) $$\mathrm{Inhibition}\;\left(\%\right)=100\times \left(\frac{\mathrm{AP}}{\mathrm{AD}-1}\right)$$

where: AP—absorbance of the test sample; AD—absorbance of control.

### 3.2. Statistical Analysis

Statistical analysis of observed results was carried out by following the method given by Kaushik et al. [29]. The standard error mean was calculated by using Microsoft excel office, 2016, and the statistical difference was calculated using one-way ANOVA (analysis of variance) and the comparison between mean was calculated by difference value. A descriptive statistical tool was used to determine the standard error means.

## 4. Conclusions

The evaluation of the antimicrobial and anti-inflammatory activity of modified solvent evaporation assisted extract of *Citrus nobilis* peel was carried out and it was observed that the extract showed significantly (*p* < 0.05) higher antimicrobial activity against Gram-positive bacteria *Staphylococcus aureus*. The extract also revealed effective anti-inflammatory activity during human red blood cell membrane stabilization (HRBC) and albumin denaturation assay. HRBC membrane is similar to lysosomal membranes and therefore is selected for in vitro evaluation. Stabilization of HRBC membrane implies that the extract may also stabilize lysosomal membranes that are essential in restricting the inflammatory response by inhibiting the release of lysosomal constituents of activated neutrophils, such as bactericidal enzymes and proteases which cause further tissue inflammation and damage upon extracellular release. Furthermore, BHT, BHA, and peel extract showed a non-significant (*p* > 0.05) difference in terms of percentage inhibition during the DPPH scavenging assay. The presence of secondary metabolites such as flavonoids and phenols in the extract may be responsible for the observed radical scavenging activity, which indicates the antioxidant potential of the extract. Additionally, the extract showed higher total flavonoid contents in comparison with other bioactive compounds, and spectra obtained from FTIR analysis confirmed the presence of functional groups hydroxyl group, amines, amides, and diketones. The LCMS analysis was performed to identify the different bioactive compounds present in the extract. Therefore, it can be concluded from the present study that although it is discarded as a waste product, *Citrus nobilis* peel, due to the presence of bioactive compounds responsible for varied biological activity, can be used as potential nutraceutical products and food supplements.

## Figures and Tables

**Table 1:** Identification of compounds present in modified solvent evaporated extract of *Citrus nobilis* peel.

Molecular Weight (g)	Compound Detected
144	Limonene
166	4-Butoxy phenol
182	3-(1-Piperidinyl methyl) phenol
203	Anthocyanin
343	Eupatorine
373	Sinensetin/ Tangeretin
403	Nobiletin
425	Vitexin/Iso-vitexin

## Data Availability

Data sharing is not applicable to this article.

## Sample Availability

Not available.
